# Supplementary material for: The conserved QTQTX motif in the SARS-CoV-2 spike protein is dispensable for cleavage and lung cell entry of the emerging variant BA.3.2
Source: J Virol. 2026 Aug 5;100(9):e00691-26. doi: 10.1128/jvi.00691-26 (PMC13595939; doi:10.1128/jvi.00691-26)
Supplement: Supplementary file 1 [file jvi.00691-26-s0001.pdf]

**Figure S1**

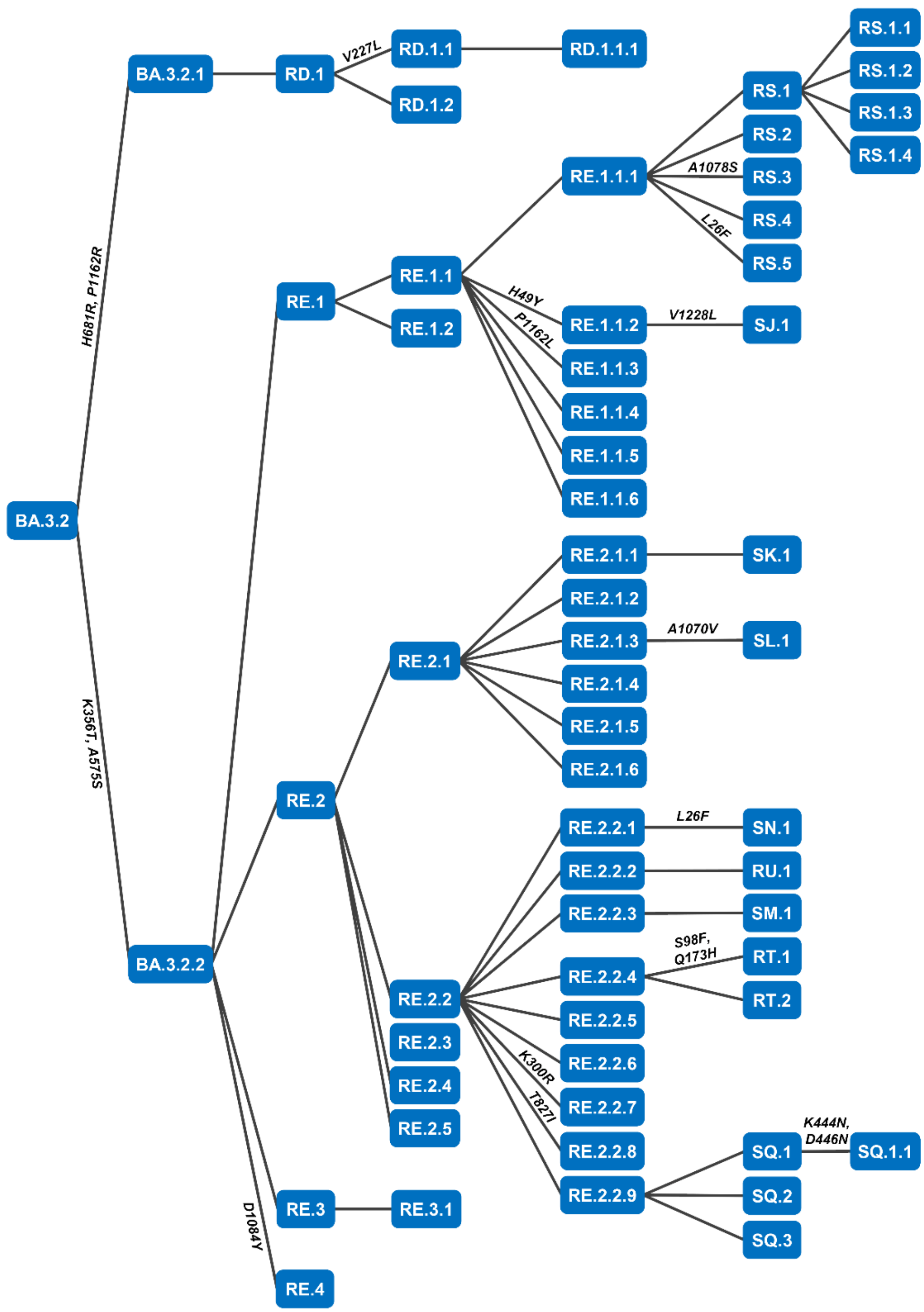


**SUPPLEMENTARY FIG S1. BA.3.2 subvariant diversification**. Evolutionary relationship of BA.3.2 subvariants with S protein mutations indicated.

## Figure S2

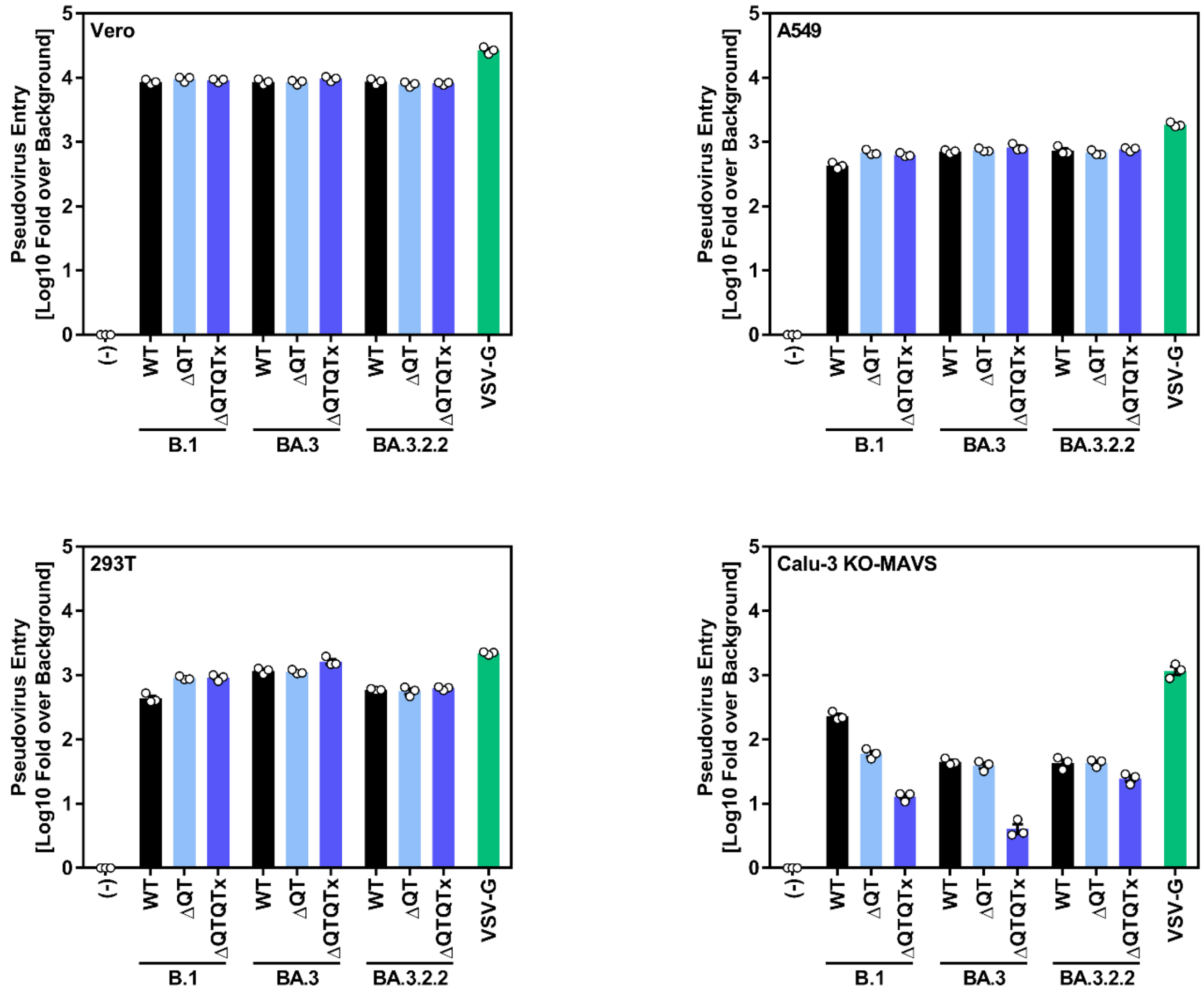


**SUPPLEMENTARY FIG S2. Cell entry of pseudovirus particles bearing the S proteins under study.** Pseudovirus entry data presented in Fig. 3B normalized to the assay background (signals obtained for control particles bearing no viral glycoprotein) and including control particles bearing no viral glycoprotein (negative control) or VSV-G (positive control).

## Figure S3

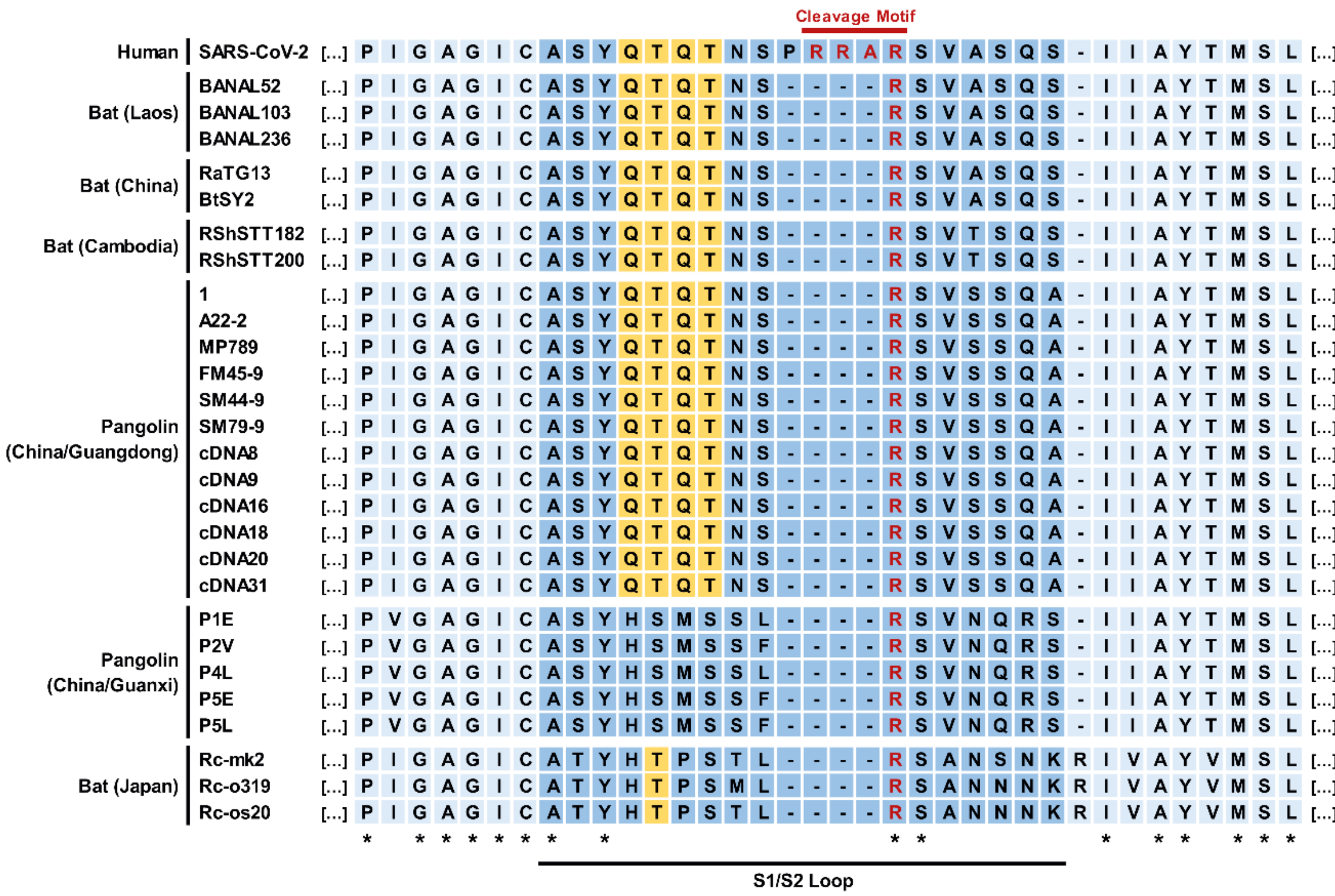


**SUPPLEMENTARY FIG S3. Several RBD clade 1b sarbecoviruses possess a QTQTx motif in their S1/S2 loop.** Sequence alignment of S1/S2 loop sequences of RBD clade 1b sarbecoviruses. Alignments were done using the Multiple Sequence Alignment by CLUSTALW web tool (https://www.genome.jp/tools-bin/clustalw) with standard parameters. Asterisks indicate conserved amino acid residues.

**Figure S4**

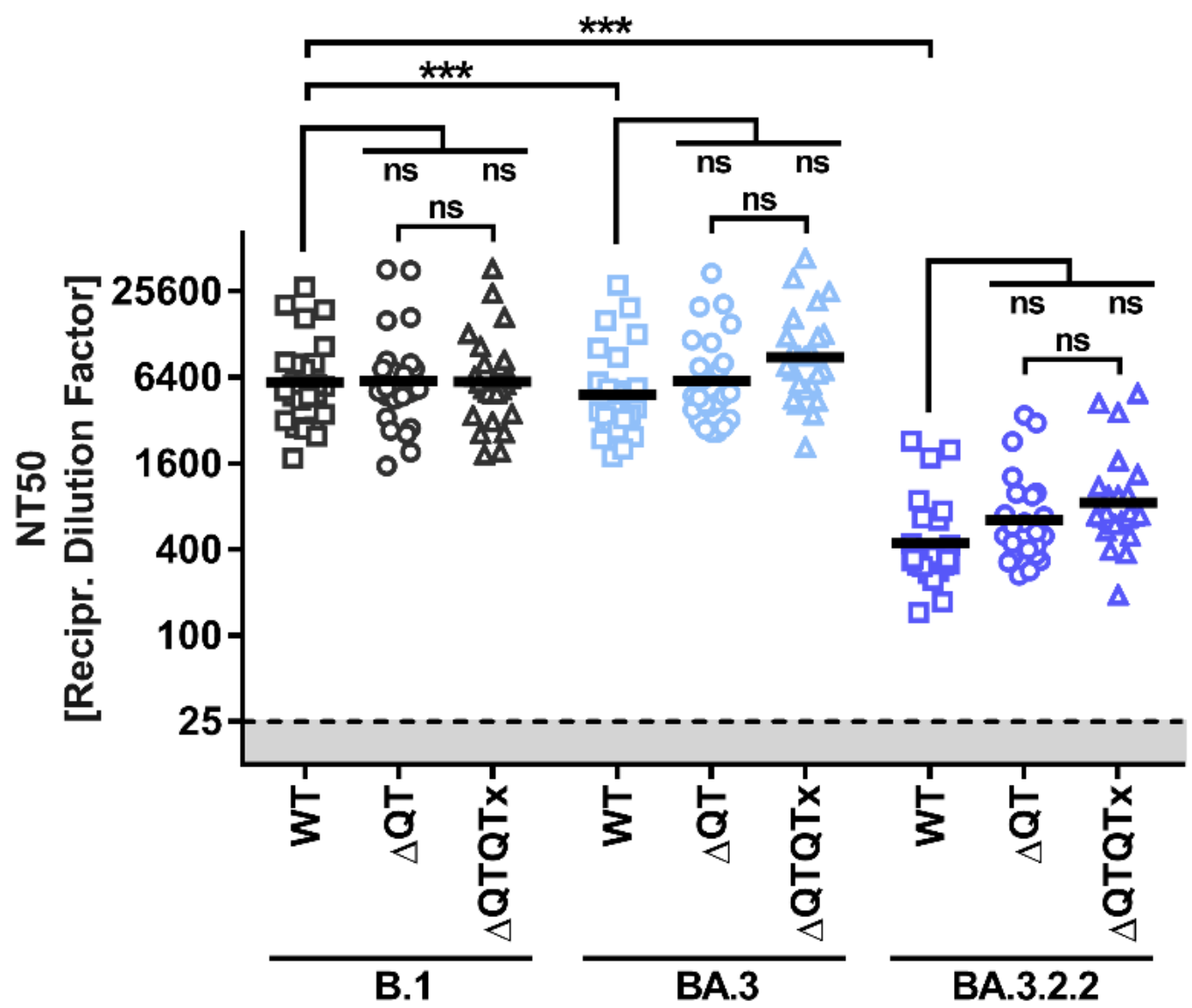


**SUPPLEMENTARY FIG S4. Neutralization sensitivity is not affected by the integrity of the QTQTx motif.** Pseudoviruses bearing the indicated S proteins were preincubated (30 min at room temperature) with serial dilutions of post-vaccination plasma or medium without plasma (control), before mixtures were added to Vero cells. Pseudovirus entry efficiency was analyzed at 16-18 h postinoculation and the relative inhibition of entry was calculated using entry of particles incubated in the absence of plasma as reference (0% inhibition). Finally, neutralizing 50 titer values indicating half-maximal inhibition of pseudovirus entry by the respective plasma were calculated using a non-linear regression model. The results from a single experiment with four technical replicates are shown. Horizontal lines indicate the geometric mean titers. Statistical significance was assessed by Kruskal-Wallis test with Dunn's multiple comparisons test (ns, p >0.05; *, p ≤0.05; **, p ≤0.01; ***, p ≤0.001).

# Figure S5

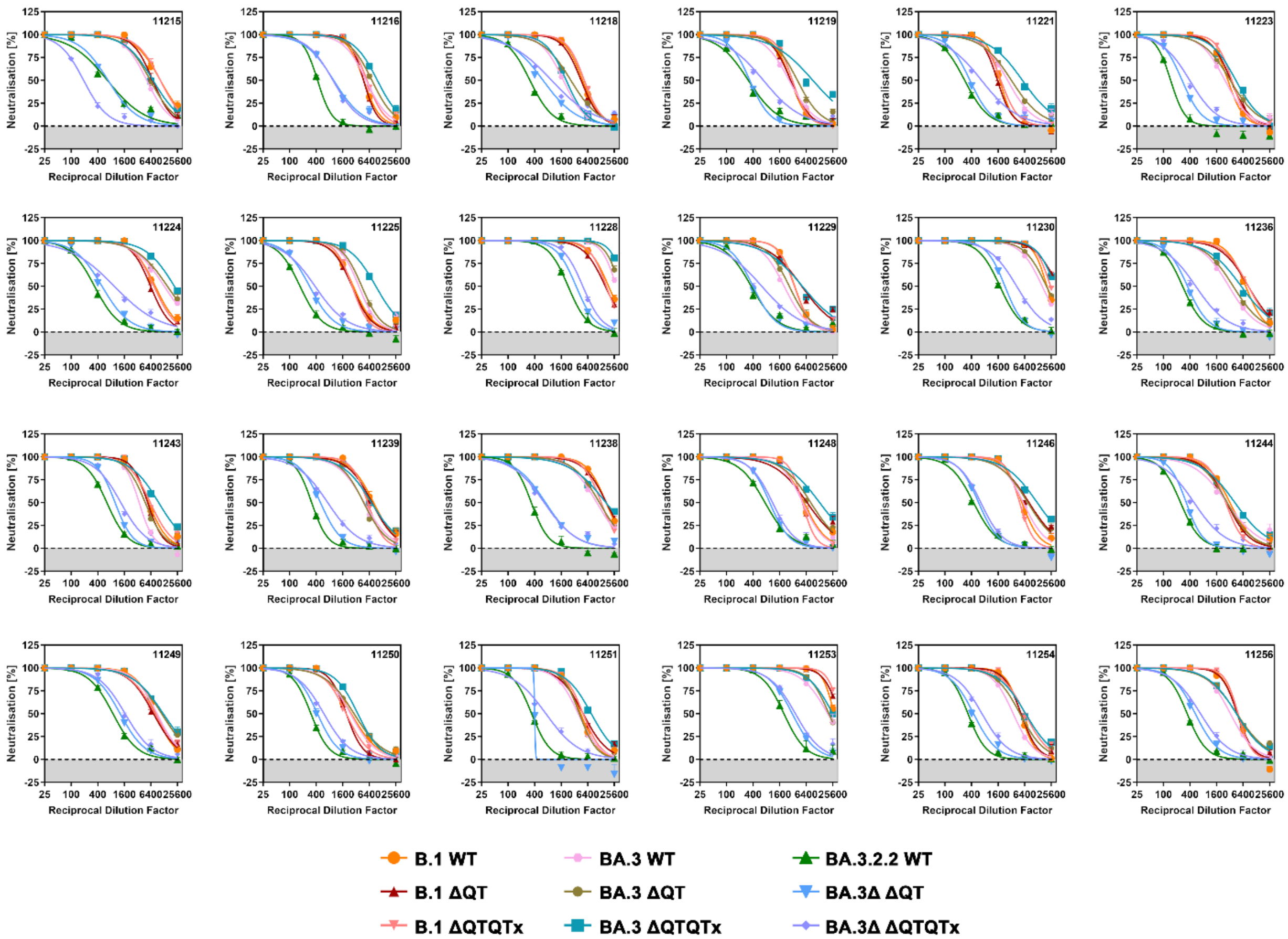


**SUPPLEMENTARY FIG S5. Neutralization of pseudovirus particles bearing the S proteins under study.** Individual neutralization data for each plasma sample
